# Accuracy of Fully Guided Implant Placement Using Bone-Supported Stackable Surgical Guides in Completely Edentulous Patients—A Retrospective Study

**DOI:** 10.3390/jcm15020652

**Published:** 2026-01-14

**Authors:** Roko Bjelica, Igor Smojver, Luka Stojić, Marko Vuletić, Tomislav Katanec, Dragana Gabrić

**Affiliations:** 1 Department of Oral Surgery, School of Dental Medicine, University of Zagreb, 10000 Zagreb, Croatia; rbjelica@sfzg.hr (R.B.); mvuletic@sfzg.hr (M.V.); tkatanec@sfzg.hr (T.K.); dgbaric@sfzg.hr (D.G.); 2 Private Practice Dentalis Art, 10000 Zagreb, Croatia; 3 Private Practice DentA Centar, 10000 Zagreb, Croatia; lukastojic@gmail.com; 4 Department of Dental Medicine, Clinical Hospital Centre Zagreb, 10000 Zagreb, Croatia

**Keywords:** dental implants, surgery, computer-assisted, printing, three-dimensional, jaw, edentulous

## Abstract

**Background/Objectives**: Precise implant positioning is critical for successful prosthetic rehabilitation, particularly in completely edentulous patients where anatomical landmarks are lost. The aim of this study was to assess the accuracy of implant placement in the edentulous maxilla and mandible using computer-assisted planning and a bone-supported stackable surgical guide protocol. **Methods**: This retrospective clinical study included 15 completely edentulous patients who received a total of 60 implants. A dual-scan protocol was utilized for planning. The surgical protocol involved a base guide fixed to the bone with pins, serving as a rigid foundation for stackable components used for osteotomy and implant insertion. Postoperative CBCT scans were superimposed onto the preoperative plan to calculate angular deviations, 3D linear deviations at the implant neck and apex, and depth deviations. **Results**: The analysis demonstrated high accuracy with a mean angular deviation of 1.25° ± 0.80°. The mean 3D linear deviation was 0.96 ± 0.57 mm at the implant neck and 1.07 ± 0.56 mm at the apex. Depth deviation showed a mean discrepancy of 0.37 ± 0.58 mm. All measured parameters were statistically significantly lower (*p* < 0.05) than the pre-established clinical safety thresholds. **Conclusions**: Within the limitations of this study, the bone-supported stackable surgical guide protocol proved to be a highly accurate method for full-arch rehabilitation. By eliminating mucosal resilience and ensuring rigid fixation, this approach enables predictable implant placement and facilitates the passive fit of screw-retained bar-supported prostheses, representing a reliable alternative to dynamic navigation in daily clinical practice.

## 1. Introduction

The implant prosthetic rehabilitation of completely edentulous patients represents a significant challenge in contemporary oral surgery, aiming to restore both function and aesthetics [1]. The concept of prosthetically driven implant placement has become the gold standard, ensuring the predictable outcomes of both surgical and prosthetic therapy [2]. This approach relies heavily on the digitization of the patient’s anatomy through the fusion of Cone-Beam Computed Tomography (CBCT) data and intraoral or laboratory surface scans, creating a “virtual patient” for precise preoperative planning [3]. Within this digital workflow, static computer-aided implant surgery (s-CAIS) has emerged as a crucial tool, offering significant advantages over free-hand implant placement, including reduced duration of surgery, minimized surgical trauma, and improved protection of vital anatomical structures [4].

However, despite the technological advancements in Computer-Aided Design/ Computer-Aided Manufacturing (CAD/CAM), discrepancies between the virtual surgical plan and the resulting clinical implant position remain a clinical reality [5]. For instance, Tahmaseb et al. [3] reported a mean deviation of 1.2 mm at the entry point and 1.4 mm at the apex, with an average angular error of 3.5°. Moreover, in fully edentulous cases utilizing mucosa-supported guides, angular deviations have been reported to reach as high as 8.4° in the maxilla, highlighting the impact of tissue resiliency on surgical precision. These deviations are rarely the result of a single factor but rather the summation of cumulative errors occurring at various stages of the workflow-from image acquisition and segmentation to 3D printing tolerances and the surgical procedure itself [6]. While s-CAIS generally demonstrates high accuracy, the recent literature consistently highlights that the nature of supporting tissues for the surgical guide is a determinant factor in the final accuracy [6].

In partially edentulous cases, tooth-supported guides provide stable and reliable seating, resulting in the highest accuracy [7]. Conversely, completely edentulous cases are often associated with a unique set of challenges due to the absence of stable dental reference points and the variable resilience of the mucosa. Mucosa-supported guides, while less invasive, are prone to intraoperative movement and tilting due to soft tissue compressibility, which can lead to significant deviations, particularly in the posterior mandible [8]. Such inaccuracies can compromise the passive fit of the final prosthesis, especially in complex rehabilitations involving screw-retained restorations [9].

To overcome the limitations of mucosal resiliency and ensure rigid fixation during surgery, bone-supported stackable surgical guides have been introduced [10]. These systems utilize a multi-component design with a base template fixed directly to the bone using osteosynthesis pins after flap elevation, providing a rigid foundation for subsequent stackable components used for bone reduction and guided implant placement [11]. Although this approach requires a more invasive surgical protocol compared to flapless mucosal supported guides, studies suggest that the elimination of guide mobility significantly enhances the transfer accuracy of the virtual plan. Nevertheless, comprehensive data quantifying the precision of these complex, stackable systems in completely edentulous patients are still needed to define clinically relevant safety margins and validate their use in daily practice [12].

The aim of this retrospective study was to assess the accuracy of implant placement in the edentulous maxilla and mandible using computer-assisted planning and a bone-supported stackable surgical guide. The primary objective was to determine the magnitude of angular and linear deviations—specifically at the implant shoulder and apex—and to evaluate whether these deviations fall within clinically acceptable limits for the fabrication of precise, bar-supported implant prostheses.

## 2. Materials and Methods

### 2.1. Study Design and Patient Selection

This retrospective clinical study included 15 completely edentulous patients (10 mandibular and 5 maxillary arches) treated between November 2024 and June 2025. The study was conducted according to the guidelines of the Declaration of Helsinki and approved by the Institutional Ethics Committee of the School of Dental Medicine, University of Zagreb (protocol code: 003-01/25-05/04, approved date: 14 June 2025). Written informed consent was obtained from all patients involved in the study after they were provided with a detailed description of the treatment protocol.

Inclusion criteria were: (1) patients aged ≥ 21 years; (2) complete edentulism in at least one arch; (3) adequate bone volume allowing for the placement of implants without simultaneous major bone augmentation; (4) requirement for alveolar ridge reduction to ensure adequate prosthetic space and sufficient bone width for long-term peri-implant tissue stability; and (5) implant rehabilitation performed using a bone-supported, stackable static surgical guide protocol. Exclusion criteria included uncontrolled systemic diseases (ASA III/IV), history of head and neck radiation, bisphosphonate therapy, and untreated periodontal disease in the opposing dentition.

### 2.2. Preoperative Planning and Prosthetic Workflow

A dual scan protocol was utilized for all patients to ensure precise transfer of the anatomical structures and prosthetic setup into the virtual model. First, the patient’s existing denture with adequate fit and occlusion was scanned using a laboratory scanner to generate a standard tessellation language (STL) file. The denture was subsequently 3D-printed with Straumann P20+ (Institut Straumann AG, Basel, Switzerland) printer using the same material intended for the surgical guide (P Pro Surgical Guide Clear, Institut Straumann AG, Basel, Switzerland). Eight spherical radiopaque markers were embedded into the denture.

**Figure 1:**
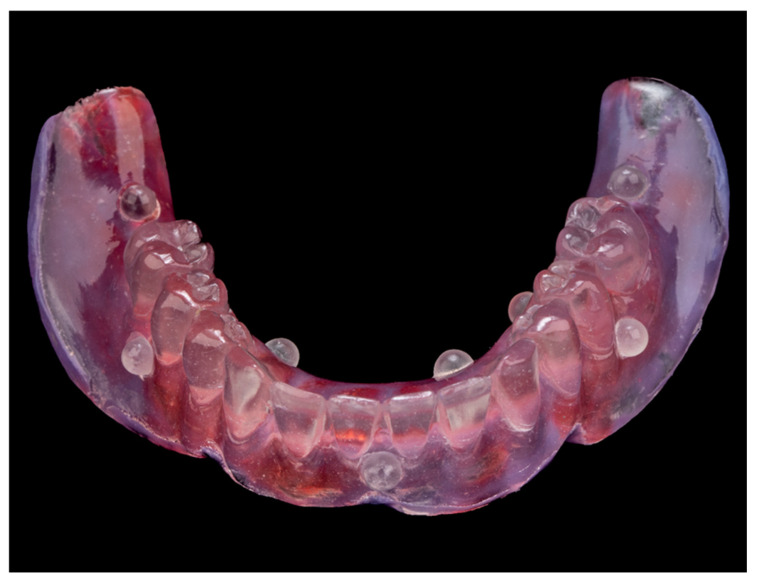
3D-printed denture with embedded radiopaque markers and polyether impression material.

Subsequently, polyether impression material (3M Impregum Penta, 3M Deutschland GmbH, Seefeld, Germany) was placed inside the denture (Figure 1) and the patient underwent a CBCT scan (Orthophos SL, Dentsply Sirona, Bensheim, Germany) while wearing the radiographic template (printed denture with markers). The scanning settings were: tube voltage of 85 kV, tube current of 6 mA, exposure time of 14.4 s, and a cylindrical field of view (FOV) of 8 cm × 8 cm. The reconstructed voxel size was 160 µm. A second scan of the prosthesis alone was performed to acquire the marker positions. The Digital Imaging and Communications in Medicine (DICOM) data from the patient scan and the STL file of the denture were imported into the planning software (coDiagnostiX^®^ version 10.9.6, Dental Wings Inc., Montreal, QC, Canada). The datasets were superimposed using radiopaque markers as reference points, creating a “virtual patient” model.

### 2.3. Virtual Planning and Guide Fabrication

Implant positions were planned according to the prosthetically driven concept, targeting a screw-retained bar-supported overdenture for all cases. A total of 60 implants (Straumann SLActive BLC or TLX, Institut Straumann AG, Basel, Switzerland) were planned. All implants were intentionally oriented vertically to facilitate the passive fit of the subsequent bar structure.

**Figure 2:**
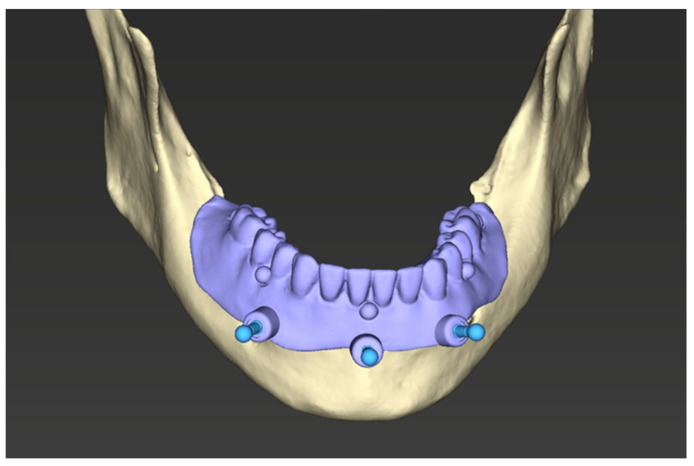
Base guide with stabilization pins.

**Figure 3:**
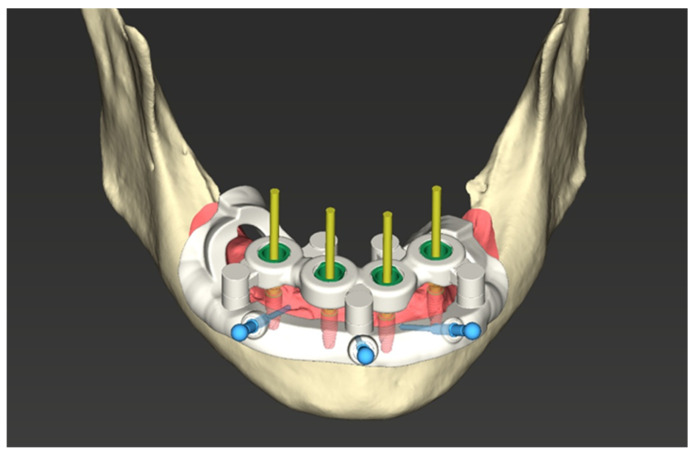
Stackable guide design with implant site preparation guide with T-sleeves.

A bone-supported stackable surgical guide system was designed. The assembly consisted of:

1. Base guide: Fixed directly to the bone with stabilizing pins (Figure 2).
2. Stackable components: Magnetically attached attachments (First4magnets, Neodymium Magnet 0.54 kg) for bone reduction (osteotomy) and implant site preparation with T-sleeves (Institut Straumann AG, Basel, Switzerland) (Figure 3).

The guides were fabricated on a 3D printer (Straumann P20+, Institut Straumann AG, Basel, Switzerland) using biocompatible resin (P Pro Surgical Guide Clear, Institut Straumann AG).

### 2.4. Surgical Protocol

**Figure 4:**
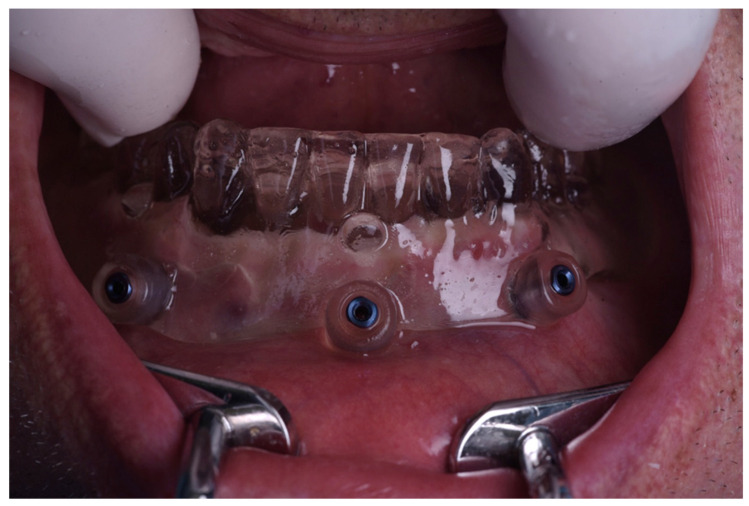
Base guide with stabilization pin holes positioned intraorally.

All surgical procedures were performed by a single specialist of oral surgery. The pharmacological protocol included antibiotic therapy with amoxicillin/clavulanic acid (875 mg + 125 mg; Klavocin bid, Pliva, Zagreb, Croatia), initiated twice daily for one day prior to surgery and continued for six days postoperatively. Following local anesthesia (4% articaine with epinephrine 1:200,000), the base guide was initially positioned over the mucosa to guide the drilling of the fixation pin channels (Figure 4). A full thickness mucoperiosteal flap was then elevated, and the surgical (primary) guide was rigidly fixed to the alveolar bone using stabilizing pins to ensure stability and eliminate mucosal resilience errors.

**Figure 5:**
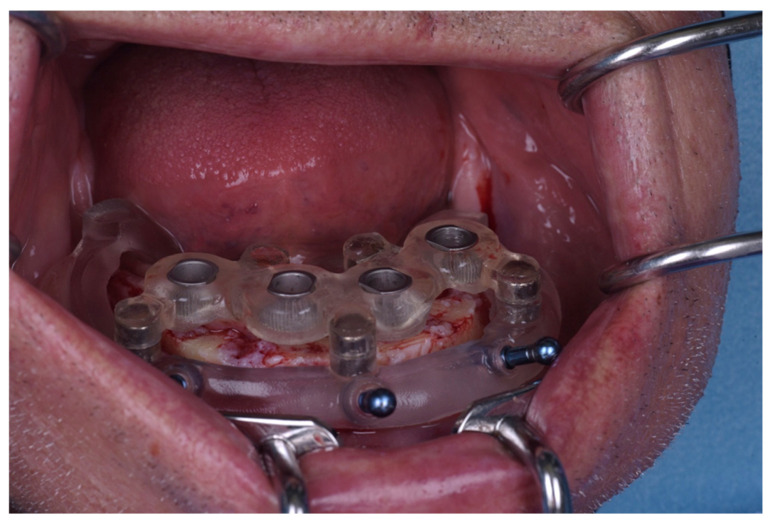
Implant site preparation (drilling) guide stacked on the primary guide.

The reduction in the alveolar ridge was executed using the magnetically attached bone-reduction guide. This step was performed to ensure sufficient vertical prosthetic space for the planned screw-retained bar-supported overdenture. The narrow, resorbed coronal portion of the ridge was removed, resulting in a wider and flatter basal bone platform. Subsequently, the drilling guide was stacked onto the primary guide (Figure 5), and the implant sites were prepared according to the specific drilling sequence. Implants were inserted through the guide to the planned depth. Flaps were sutured using non-resorbable monofilament sutures.

Immediate postoperative care included a single intramuscular dose of 8 mg dexamethasone to minimize edema. Non-steroidal anti-inflammatory drugs (NSAIDs) were prescribed for the first three postoperative days for pain management. Additionally, patients were instructed to rinse with 0.12% chlorhexidine (Curasept ADS 212, Curaden AG, Kriens, Switzerland) twice daily for ten days to maintain oral hygiene. Final rehabilitation in all cases consisted of a bar-supported overdenture.

### 2.5. Accuracy Evaluation

Postoperative CBCT scans were obtained after the healing period using the same acquisition parameters as the preoperative scans, following the ALARA (As Low As Reasonably Achievable) principles. The postoperative DICOM data were imported into the coDiagnostiX^®^ (version 10.9.6) software and superimposed onto the preoperative planning data. The registration process utilized an automated surface best-fit matching algorithm (iterative closest point algorithm) within the treatment evaluation module of coDiagnostiX^®^. The process was performed twice by a single experienced investigator to assess intra-observer reliability.

**Figure 6:**
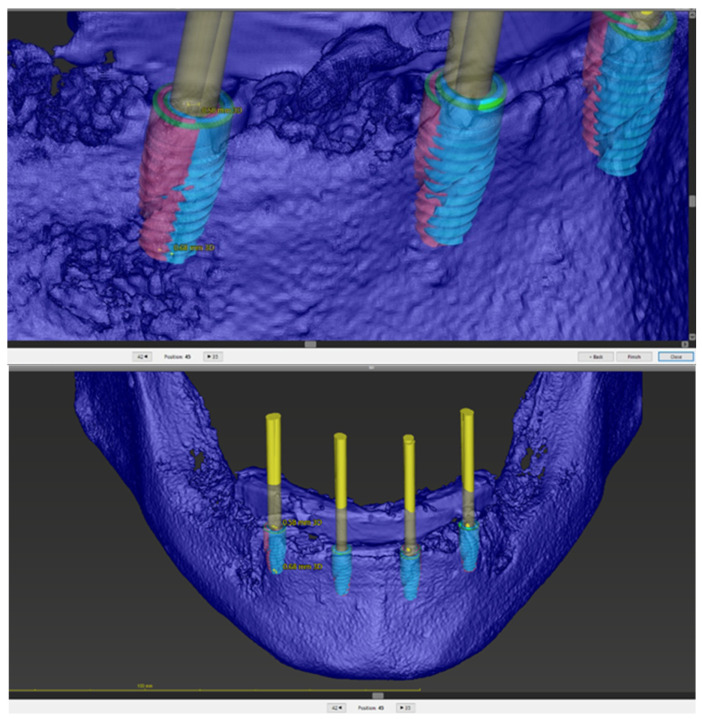
Accuracy evaluation within the treatment evaluation module of coDiagnostiX^®^, showing the 3D superimposition of the planned (blue) and actual (red) implant positions.

**Figure 7:**
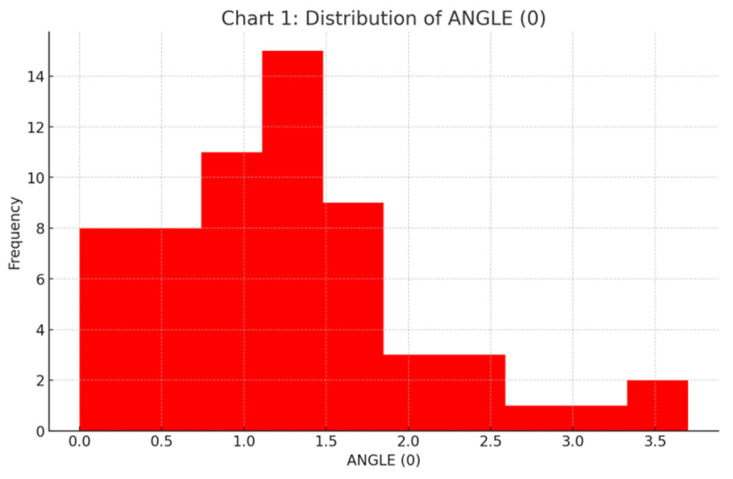
Frequency distribution histogram for angular deviation, illustrating the concentration of implants within the clinical safety threshold of 3.5°.

The deviation between the planned and actual implants was calculated automatically by the software. Four deviation parameters were analyzed for each implant (Figure 6 and Figure 7):

- 3D deviation at the implant neck: The linear distance (mm) between the center of the implant platform of the planned and actual implant.
- 3D deviation at implant apex (tip): The linear distance (mm) between the center of the implant apex of the planned and actual implant.
- Depth deviation: The vertical discrepancy (mm) along the longitudinal axis of the implant.
- Angular deviation: The three-dimensional angle (degrees) between the longitudinal axes of the planned and actual implant.

### 2.6. Statistical Analysis

Descriptive statistics (mean, standard deviation, median, interquartile range [IQR], minimum, and maximum) were calculated for all deviation parameters. The normality of the data distribution was assessed using the Kolmogorov–Smirnov test.

To evaluate clinical acceptability, a one-sample *t*-test was performed to test the hypothesis that the observed mean deviations were significantly lower than the following clinically relevant reference thresholds: 3.5° for angular deviation, 2.5 mm for 3D apical deviation, 2.0 mm for 3D deviation at the implant neck, and 2.0 mm for depth deviation. The threshold for clinically acceptable accuracy was set in accordance with the recommendations of the 5th ITI Consensus Conference [13]. A *p*-value of <0.05 was considered statistically significant. All analyses were performed using MedCalc^®^ Statistical Software version 20.023 (MedCalc Software Ltd., Ostend, Belgium).

## 3. Results

A total of 60 implants were placed in 15 completely edentulous patients using bone-supported stackable guides. No intraoperative complications were recorded, and all guides achieved rigid fixation.

The descriptive statistics for all deviation parameters are summarized in Table 1. The descriptive differences between maxilla and the mandible are summarized in Table 2.

The quantitative analysis demonstrated high accuracy across all measured dimensions. The one-sample *t*-test confirmed that the mean deviations for all parameters were statistically significantly lower than the pre-established clinical safety thresholds (*p* < 0.05).

The mean angular deviation was 1.25° ± 0.80° (Median: 1.20°; IQR: 0.90). This value was significantly below the 3.5° clinical reference threshold indicating precise control over implant trajectory (Figure 7).

**Figure 8:**
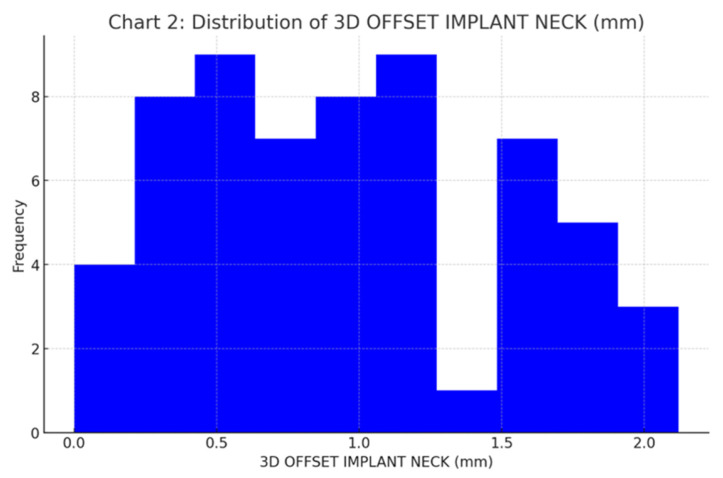
Frequency distribution histogram for 3D linear deviation at the implant neck (mm).

The mean 3D deviation at the implant neck was 0.96 ± 0.57 mm (Median: 0.86 mm), which proved to be significantly lower than the 2.0 mm safety limit (Figure 8).

**Figure 9:**
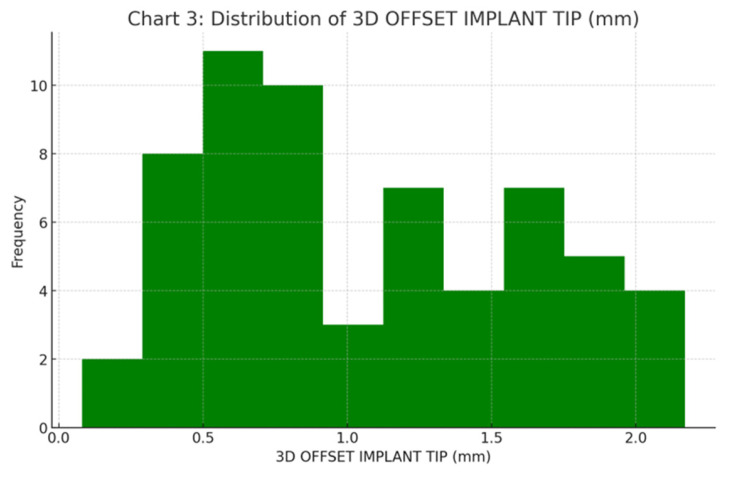
Frequency distribution histogram for 3D linear deviation at the implant tip (mm).

The mean 3D apical deviation was 1.07 ± 0.56 mm (Median: 0.87 mm). Despite the increased distance from the guide sleeve, the apical position remained well within the 2.5 mm acceptance threshold (Figure 9).

**Figure 10:**
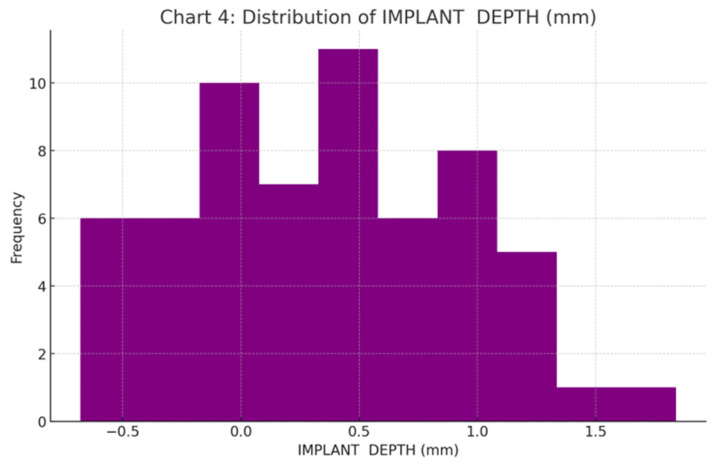
Frequency distribution histogram for vertical (depth) deviation (mm), where positive and negative values represent over- and under-drilling relative to the plan.

The vertical (depth) deviation showed the lowest mean discrepancy of 0.37 ± 0.58 mm. This was well within the 2.0 mm limit, confirming the effectiveness of the stackable guide’s stops (Figure 10).

Overall, the narrow interquartile ranges (IQR) observed across all variables suggest a homogeneous performance of the stackable guide system.

## 4. Discussion

The primary objective of this retrospective study was to evaluate the clinical accuracy of a bone-supported, stackable static guide protocol for the rehabilitation of completely edentulous patients. The presented results showed a high level of precision, with a mean global linear deviation of 0.96 ± 0.57 mm at the implant neck and 1.07 ± 0.56 mm at the apex. Furthermore, the mean angular deviation was restricted to 1.25 ± 0.80°. The observed values were statistically significantly below clinical safety thresholds, ensuring the protection of vital structures such as the inferior alveolar nerve and maxillary sinus. The narrow interquartile ranges observed across all parameters suggest that the stackable guide protocol offers a highly reproducible workflow, minimizing the “outliers” often associated with conventional computer-assisted implant surgery (s-CAIS) in edentulous cases [14].

A critical finding of the present study is the high level of control achieved with stackable bone-supported guides compared to historical data available in the literature for mucosa-supported guides [15]. In completely edentulous patients, the resilience of the soft tissues has traditionally been the main confounding factor for accuracy [16]. In a systematic review by Marlière et al. [4], mean angular deviations for mucosa-supported guides were reported to range between 1.85° and 8.4°. Specific clinical studies underscore this issue, for instance, Arisan et al. [17] reported a mean angular deviation of 4.6° and linear deviations often exceeding 1.5 mm when using mucosa-supported stereolithographic guides, attributing these errors to the inherent mobility of the soft tissue during drilling the implant site. Similarly, Vasak et al. [15] found that mucosal thickness significantly correlates with deviations, as thicker tissue allows for greater guide displacement.

In contrast, the protocol used in the presented study utilized a bone-supported base fixed with stabilization pins. By flap elevation and seating the guide directly on the bone, the variable mucosal resilience was effectively eliminated. This approach aligns with the findings of Ozan et al. [18], who compared three types of support and concluded that rigid support (tooth or bone) significantly outperforms mucosa-supported designs in terms of linear accuracy. While flapless surgery is often advocated for reduced morbidity, as noted by Naeini et al. [6], the data obtained in this study suggests that for fully edentulous arches, the “open-flap” bone-supported approach provides a biomechanical stability that is paramount for precise screw-retained prosthetic rehabilitation, outweighing the benefits of a flapless approach in complex cases.

The stackable or multilayer nature of the surgical guide system utilized in this study represents a significant progression from single-layer templates. In this workflow, the base template remains fixed to the bone throughout the surgery, serving as a stable anchorage for subsequent, interchangeable components (bone reduction and implant site preparation guides). This concept is supported by the recent study by Levy-Bohbot et al. [19], which reported a 96.5% implant survival rate and high prosthetic success using a similar stackable protocol for immediate full-arch loading.

The stability provided by the stackable design addresses a limitation highlighted by Wang et al. [5], who reported angular deviations of 7.14° in partially guided protocols where the guide is removed prior to implant insertion. By maintaining physical guidance through the implant site preparation and insertion phases via stackable components, the protocol in the presented study minimizes the cumulative error transfer, which is also described by D’Haese et al. [8]. Furthermore, Cristache et al. [20] recently demonstrated that a full digital workflow using stackable guides allows for immediate fixed rehabilitation with high predictability, reporting angular deviations of 2.67°. The presented results (1.25 ± 0.80°) compare favorably even to these recent findings, possibly due to the specific locking mechanism of the stackable components used in this study which prevents micro-movements between the guides.

With the rapid advancement of dynamic computer-aided implant surgery (d-CAIS) and robotic systems, it is necessary to contextualize and the performance of static guides and compare them with d-CAIS. A recent systematic review by Wei et al. [21] analyzed the accuracy of dynamic navigation and reported a pooled mean angular deviation of 3.59° (95% CI: 2.09–5.09) and a global apex deviation of 1.33 mm. Similarly, Block et al. [22] reported angular deviations of approximately 3.25° in a large clinical series using dynamic navigation. While the current study observed numerically lower mean angular deviations (1.25°), it is crucial to interpret this difference with caution. Dynamic navigation offers the distinct advantage of real-time visualization and the ability to intraoperatively adjust the implant position, which static guides do not allow. However, d-CAIS systems are surgeon-sensitive and require a learning curve to manage hand-eye coordination, as noted by Pellegrino et al. [23]. On the other hand, the stackable static guide offers a physical “stop” and rigid trajectory control, which may reduce errors during full-arch osteotomies. Therefore, while robotic systems, which Khaohoen et al. [10] found to have the lowest deviations (0.81 mm coronal), represent the future of automation, the bone-supported stackable guide remains a highly competitive, cost-effective, and precise solution for full-arch rehabilitation in current clinical practice.

From a clinical perspective, the statistical significance of deviations is less important than their clinical consequences. The 6th ITI Consensus Conference recommends a safety margin of 2 mm to avoid damage to vital anatomical structures [24]. In this study, the maximum observed apical deviation was 2.17 mm, with a mean of just 1.07 mm. This indicates that the stackable guide system consistently performs well within the safe zones required for avoiding the vital anatomical structures. Moreover, the low angular deviation is clinically decisive for the fabrication of the bar-supported overdentures used in this study. High angular discrepancies in multi-implant cases can lead to a lack of passive fit, inducing stress on the implants and prosthetic components [25]. As concluded by Jreige et al. [26], the integration of a precise digital workflow from planning to execution is essential for minimizing these prosthetic complications [27]. The achieved accuracy facilitates the passive fit of immediate restorations and minimizes chairside adjustment time, consistent with the findings of Schnutenhaus et al. [27].

This study has limitations inherent to its retrospective design. The sample size, although sufficient for statistical power regarding accuracy variables, is relatively small. Furthermore, the absence of a control group represents a primary methodological constraint. As this was a retrospective clinical analysis, a direct comparison with other modalities—such as mucosa-supported guides or dynamic navigation (d-CAIS)—was not performed. Consequently, while the presented results compare favorably to historical data in the literature regarding mucosal resilience errors, the degree to which the bone-supported approach offers a superior accuracy-to-morbidity ratio compared to less invasive protocols cannot be statistically concluded. The study did not evaluate Patient-Reported Outcome Measures (PROMs) such as pain or satisfaction, which is a notable limitation considering the invasiveness of the required full-thickness flap and the need for stabilization pins. Consequently, it remains unclear whether the achieved surgical precision and passive prosthetic fit sufficiently compensate for the potentially increased postoperative discomfort and surgical trauma associated with this bone-supported approach. Additionally, all surgeries were performed by a single experienced surgeon, which may limit the generalizability of the findings to less experienced clinicians. As indicated by Cassetta et al. [28], the learning curve can influence the accuracy of guided surgery. While an automated best-fit algorithm was utilized for superimposition, potential registration errors remain an inherent limitation in CBCT-based accuracy studies and may represent a source of measurement bias. Future multicenter randomized controlled trials comparing stackable guides directly with dynamic navigation in edentulous arches would be valuable to further confirm these findings.

## 5. Conclusions

Within the limitations of this retrospective study, the bone-supported stackable surgical guide protocol proved highly accurate for implant placement in completely edentulous patients. The presented workflow effectively minimizes inaccuracies related to mucosal resilience by utilizing rigid bone fixation. The achieved precision ensures the protection of vital structures and supports the predictable passive fit of screw-retained prostheses. Despite the emergence of dynamic navigation and robotic technologies, this protocol remains a reliable and accessible solution for full arch rehabilitation.

## Figures and Tables

**Table 1:** Descriptive statistics of deviation parameters and comparison with clinical acceptance thresholds.

Variable	Mean ± SD	Median (IQR)	Min	Max	Clinical Threshold	*p*-Value
Angular deviation (°)	1.25 ± 0.80	1.20 (0.90)	0.00	3.70	<3.5°	8.54 × 10^−30^ *
3D Deviation—Neck (mm)	0.96 ± 0.57	0.86 (0.84)	0.00	2.12	<2.0 mm	1.69 × 10^−20^ *
3D Deviation—Apex (mm)	1.07 ± 0.56	0.87 (0.96)	0.08	2.17	<2.5 mm	1.25 × 10^−27^ *
Depth deviation (mm)	0.37 ± 0.58	0.35 (0.84)	-0.68	1.84	<2.0 mm	5.84 × 10^−30^ *

* Statistically significant (*p* < 0.05).

**Table 2:** Descriptive statistics of deviation parameters in maxilla and the mandible.

Variable	Maxilla (n = 20 Implants)	Mandible (n = 40 Implants)
Angular deviation (°)	1.38 ± 0.88	1.19 ± 0.76
3D Deviation—Neck (mm)	1.05 ± 0.62	0.92 ± 0.54
3D Deviation—Apex (mm)	1.18 ± 0.64	1.01 ± 0.52
Depth deviation (mm)	0.32 ± 0.51	0.40 ± 0.61

## Data Availability

The data presented in this study are available upon request from the corresponding author.
